# Milk Sickness

**Published:** 1865-03

**Authors:** E. W. Boyles

**Affiliations:** Clay City, Illinois


### MILK SICKNESS.

By E. W. BOYLES, of Clay City, Ill.

*Messrs. Editors of the Journal:—*

By your permission I wish to communicate a few words to the readers of the *Journal*, in regard to that disease familiarly known as *Milk Sickness*, for the purpose of eliciting some information on the subject.

It makes it appearance in the fall season at the same time that cattle have, and die with, the "Trembles," or "Slows," and is peculiar to districts of country where cattle have the aforesaid disease. The Premonitory Symptoms are a general feeling of lassitude, with nausea, sometimes for several days, and even weeks, before the patient becomes confined to the bed. Next the symptoms are similar to those of Gastritis—frequent, almost constant vomiting, violent burning pain in the epigastrium, great thirst, everything taken into the stomach is almost immediately ejected. Pulse as a general thing thready and feeble, great muscular depression, coldness of the extremities. A thin brownish coat on the tongue, breath very offensive, the odor is so peculiar that a person having once *smelled* it, will recognize it ever after. Bowels always obstinately constipated in the early stages.

These are the most prominent symptoms as they generally appear, though they differ considerably in different cases, and there are often many others present, viz., hiccough, sometimes considerable fever is present, and a peculiar sighing, blowing noise on expiration, and by placing the palm of the hand on the bowels a thrill will be communicated at every diastole of the heart, exactly as though the hand was placed over the region of that organ. This symptom, I *believe* to be always present.

You will perceive that the symptoms are closely allied to those of Gastritis, but I have never noticed the brain being augmented by by pressure in this disease as it is in Idiopathic Gastritis. Again, the offensive odor that is always present in this disease is not present in Gastritis. And perhaps the most characteristic symptom is the obstinate constipation of the bowels in the early stages of the disease, which, though frequent, is not invariably the case in Gastritis.

Now, a few words in regard to treatment. When I first had to deal with this disease, after producing an evacuation of the bowels, I gave quinine, but my patients invariably grew worse under its use, so I have long since given that up. My plan of treatment now is, large doses of calomel combined with some powerful cathartic, often croton oil, when the stomach will retain it, but we can scarcely ever get enough medicine of any kind to remain in the stomach long enough to produce purging, so we resort to purgative enemata. After effecting an evacuation I then keep up the use of mercury, until the patient is slightly ptyalized, or better, and I give alcoholic stimulants with an unsparing hand. It is next to impossible to intoxicate a patient laboring under this disease. I have treated twenty cases during the last fall, eighteen of which recovered. In the cases that proved fatal dysenteric diarrhœa ensued, with much griping and tenesmus.

I have seen a few patients die before any action could be obtained on the bowels.

Now, Messrs. Editors, what is it? It is a disease about which there is great contrariety of opinion. The majority of physicians in this section of country call it Congestive Fever, but it is as far from Pernicious Fever as day is from night. Milk Sickness, I am satisfied, is a misnomer, for I have treated several cases where the patient never indulged in drinking milk or eating beef or butter, because they were afraid of contracting the disease, that being the prevalent opinion of the way it is communicated. Yet it is peculiar to districts where cattle have the "trembles."

I wish to be brief as possible, so I will close this, I fear, too lengthy article. I have given the prominent symptoms as they have appeared under my observation, together with the prominent features of my mode of treatment. I, of course, use a good many other things, as subsidiary blisters to the epigastrium, etc. I would be pleased to hear from you or any of your correspondents on the subject.

